# Efficient 3D-Silver Flower-like Microstructures for Non-Enzymatic Hydrogen Peroxide (H_2_O_2_)  Amperometric Detection

**DOI:** 10.1038/s41598-017-11965-9

**Published:** 2017-09-22

**Authors:** Gumaa A. El-Nagar, Radwan M. Sarhan, Ahed Abouserie, Natalia Maticiuc, Matias Bargheer, Iver Lauermann, Christina Roth

**Affiliations:** 1 Department of Chemistry, Faculty of Science, Cairo University, Giza, 12613 Egypt; 2 Institut für Chemie und Biochemie Physikalische und Theoretische Chemie, Freie Universität Berlin, 14195 Berlin, Germany; 3 Institute of Physics and Astronomy, University of Potsdam, Karl-Liebknecht-Strasse 24–25, 14476 Potsdam, Germany; 4 Humboldt-Universität zu Berlin, School of Analytical Sciences Adlershof (SALSA), Albert-Einstein-Str. 5–9, 10099 Berlin, Germany; 5 Institute of chemistry, University of Potsdam, Karl-Liebknecht-Strasse 24–25, Potsdam, D-14476 Germany; 6 Helmholtz-Zentrum Berlin für Materialien und Energie, Hahn-Meitner-Platz 1, 14109 Berlin, Germany

## Abstract

We present an efficient non-enzymatic hydrogen peroxide sensor composed of flower-like silver microstructures. The silver microstructures´ morphology is controlled by adding minute amounts of either succinic or malonic acid as directing agents. Morphologically, silver particles showed ball-like structures in the absence of both directing agents, while the presence of 50 ppm of succinic acid and malonic acid lead to monodisperse chrysanthemum and water-lily flower-like structure, respectively. A higher concentration of succinic acid resulted in a rose flower-like structures. Electrochemically, the rose flower-like silver microstructures exhibited the best performance for H_2_O_2_ detection as evaluated by their outstanding electrocatalytic activity (12 times higher) and sensitivity (2.4 mM^−1^ cm^−2^, 24 times higher) with lower detection limit (0.4 µM, 5 times smaller) together with their excellent H_2_O_2_ selectivity compared to that of the ball-shaped structures. Additionally, rose-flower microstructures exhibited excellent long-term stability; 11 and 3 times higher compared to ball- and water-lily structures, respectively. This substantial performance enhancement is attributed to their unique flower-like structure providing a higher number of active surface sites (at least 8 times higher) and a faster detachment rate of *in-situ* generated oxygen bubbles from their surface.

## Introduction

Hydrogen peroxide (H_2_O_2_) detection has recently attracted significant attention, thanks to its crucial role in a wide range of applications including biological systems, environmental protection together with food, pharmaceutical and clinical industries^1,2^. Thus, the development of an efficient (i.e., accurate, sensitive, selective and rapid) low-cost H_2_O_2_ sensor is extremely important. Several H_2_O_2_ detection methods have been employed such as spectrophotometry^3^, chromatography^4^, chemiluminescence^5,6^, titrimetry^7^ and electrochemistry^8–11^. Electrochemical methods using enzyme-based biosensors are the most appropriate and effective tool for H_2_O_2_ detection owing to their unique features including low cost, high selectivity, simplicity, and excellent sensitivity. However, the low stability, limited lifetime and the complicated immobilization procedures of the enzyme-based catalysts together with their susceptibility to change with the operating environment (e.g., pH, temperature, toxic chemicals) limit their practical applications^12–14^.

As a result, the development of stable and efficient H_2_O_2_ non-enzymatic sensors is urgently required. In this regard, various metal nanoparticles (e.g., AuNPs, PtNPs, PdNPs, AgNPs)^15–19^ and metal oxide nanostructures (e.g., MnO_2_, Fe_2_O_3_, etc)^20–22^ are alternatively employed. Among them, silver nanoparticles (AgNPs) have aroused increasing interest due to their unique combination of biocompatibility and highest electrical conductivity together with their  outstanding electrocatalytic activity^23,24^. But their structure agglomeration, collapse and segregation during long-term measurement results in diminished performance. Thus, the development of a catalyst with a high mechanical stability and robust structure is required for  efficient H_2_O_2_ detection.

In this paper, an efficient and stable non-enzymatic H_2_O_2_ sensor of 3D-silver microflowers with different roughness and shapes is introduced. Dicarboxylic acids (i.e., malonic and succinic acids) were used as capping agents in order to (a) stabilize the growth of silver nanoparticles, as well as  protect them from agglomeration and losing their surface features and (b) controlling their morphology and roughness via the interaction (e.g., electronic interaction, strong hydrogen-bonding, or weak van der Waals interactions) between the malonic and succinic acids functionalities (carboxylic groups) with the silver nanoparticles. Different concentrations of succinic and malonic acids were used to prepare silver microparticles with various geometries (i.e., ball and flower-like structures). The effect of the *in-situ* formed oxygen bubbles size atop of the as-prepared electrodes and their accumulation/detachment rate on the stability of the suggested electrodes are investigated, for the first time. All prepared silver microstructures are dispersed in a stable chitosan matrix to avoid their aggregation/sintering.

## Experimental

### Chemicals

Silver nitrate, succinic acid, malonic acid, uric acid, urea, glucose, potassium hydroxide, chitosan and ascorbic acid were all purchased from Sigma-Aldrich and were used as received without any further purification. All solutions were prepared using deionized water with resistivity of 18.2 MΩ cm which was prepared using a Milli-Q reagent deionizer (Millipore).

### Silver Microstructures Synthesis and Fabrication

Hierarchical silver microflowers were synthesized via a previously described procedure with a little modification^25^. Ascorbic acid was used as a reducing agent while different dicarboxylic acids were used as structure-directing agents as well as capping agents. This results in formation of silver structures with various morphologies ranging from silver microspheres to 3D silver microflowers. Briefly, 1 ml of 1 M of aqueous solution of AgNO_3_ and 50 μL of 0.25 M aqueous solution of succinic acid were mixed in 10 ml of deionized water for 10 min in an ice-water bath. To initiate the formation of microstructures, 1 ml of 1 M aqueous solution of ascorbic acid was then quickly added and vigorously stirred for 15 min. The solution color turned from colorless to dark grey immediately and finally a large quantity of the silver particles precipitated. The silver microstructures were then collected and washed several times with deionized water and dried under vacuum. This resulted in the formation of silver microstructures with chrysanthemum flower-like structures. Doubling the concentration of succinic acid resulted in rose flower-like structures. The same procedures were used to synthesize water-lily flower like structures, by replacing the succinic acid with malonic acid as a directing agent. For sake of comparison, the silver microparticles were prepared without adding a structure-directing agent. This resulted in the formation of the ball-shaped silver microstructures.

#### Electrode preparation was performed as follows

5 mg of the above obtained silver microstructure powders were dispersed into 3 ml of chitosan solution (0.5%) and sonicated for 15 minutes. Then, 10 μl of the obtained suspension was drop-cast atop of a previously mechanically cleaned glassy carbon electrode (GCE) using alumina powder and allowed to dry at room temperature. To prepare a solution of the poorly soluble chitosan, 0.5 g of chitosan was dissolved in 1% acetic acid aqueous solution overnight. The chitosan matrix is used to stabilize the silver structures so that they will resist to structure deformation and aggregation during long-term measurements.

### Electrodes Characterization

All the electrochemical tests were performed at room temperature in a conventional three-electrode glass cell using a Gamry setup potentiostat/Galvanostat, where a coiled Pt wire and a saturated calomel electrode (SCE) served as a counter and reference electrode, respectively. Electrochemical impedance spectroscopy (EIS) measurements were carried out at −0.25 V (N.B., Nyquist plot was first measured at different potentials and −0.25 V was selected as the best potential, at which the kinetic control region can be easily observed) with a disturbance potential of 5 mV and a frequency range from 1 MHz to 0.1 Hz. A scanning electron microscope coupled with an energy dispersive X-ray spectrometer (JEOL JSM-6510) was used to evaluate the electrode morphology and composition. X-ray diffraction (XRD using STOE STADI) in transmission geometry XRD was performed with Cu K_α_ radiation (λ = 1.54 Å) and position sensitive detector to identify the crystallographic structure of the as prepared silver microstructures. X-ray photoelectron spectroscopy (XPS, CLAM4 electron analyzer from Thermo VG scientific), using a Mg Kα X-ray source (1254 eV) was used to determine the samples chemical composition. For evaluation, a linear background was subtracted and peaks were fitted using Voigt functions with identical FWHM for each component of the same element.

## Results and Discussion

### Material and Electrochemical Characterizations

**Figure 1:**
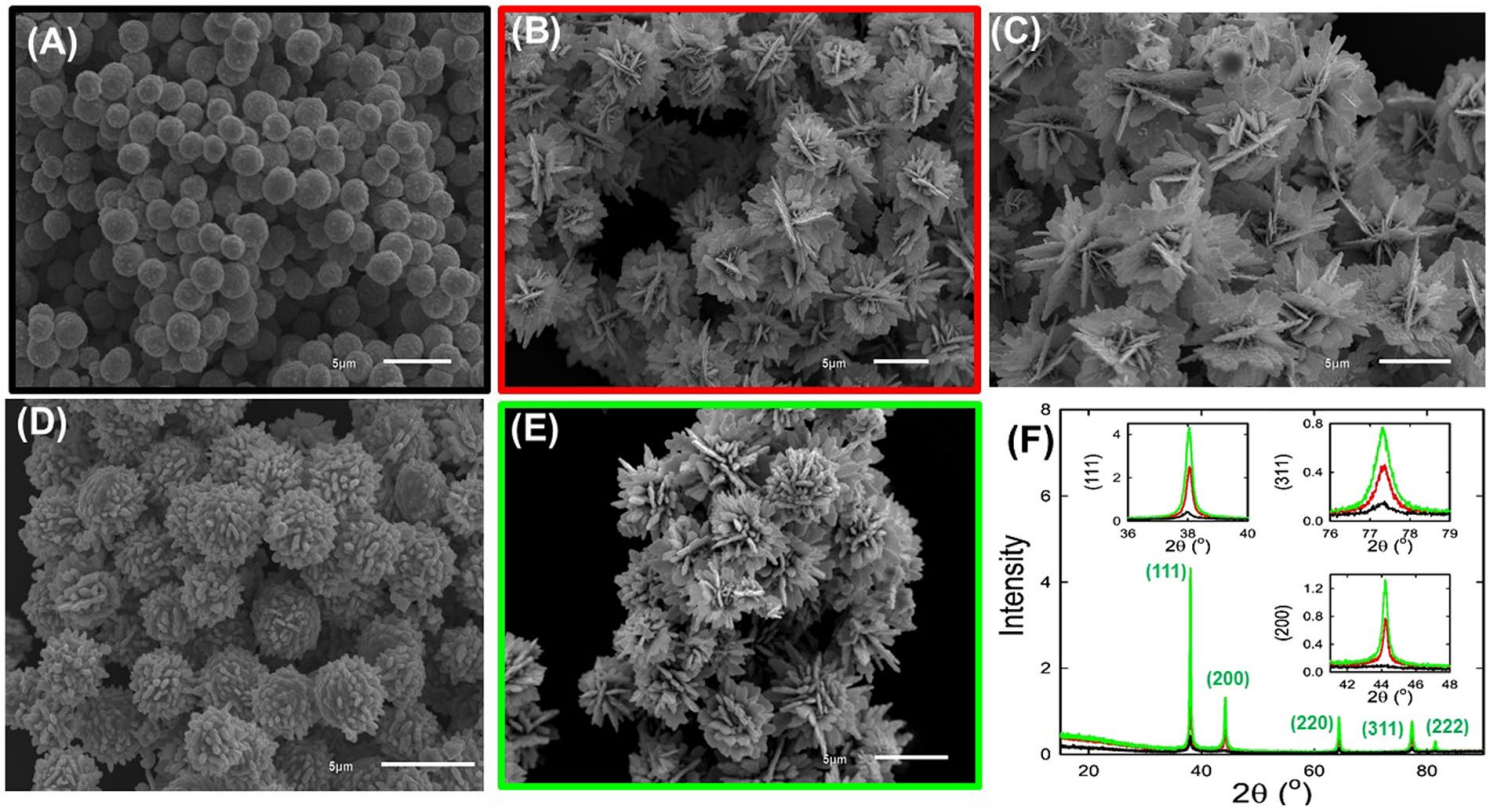
(**A–E)** SEM images of silver microstructures prepared (**A**) in the absence of directing agents and in the presence of (**B**) 50 ppm malonic acid, (**C**) 100 ppm malonic acid, (**D**) 50 ppm succinic acid and (**E**) 100 ppm succinic acid. Frames color code the graphs in panel (**F**): XRD pattern of ball (Ag_ball_, black-line), lily-flower (Ag_lily-flower_, red-line) and rose-flower (Ag_Rose-flower_, green-line) with assigned Miller indices. Insets show zooms into the (111), (200) and (311) reflections.

The morphology and structure of the as-prepared silver catalysts were examined by SEM and XRD, as seen in Fig. 1. Silver microparticles with ball-like structures were obtained  in the absence of both directing agents (i.e., malonic and succinic acids) with an average ball-diameter of 2 μm (see Fig. 1A, assigned as Ag_ball_/GCE, color code black). Interestingly, the presence of a minute amount of malonic acid (~50 ppm) resulted in the formation of monodisperse and homogenous water-lily flower-like silver structures composed of well-assembled and loosely packed silver nanosheets (Fig. 1B, denoted as Ag_lily-flower_/GCE, color code red). Using higher malonic acid concentration (~100 ppm) yielded water-lily flower - like structures as well (see Fig. 1C), but with more loosely packed silver nanosheets compared to that prepared with 50 ppm solution. We only discuss the electrocatalytic behavior of the water-lily flowers prepared by 50 ppm, since they yield a better electrocatalytic performance attributed to their higher electroactive surface area originating from their higher amount of loosely packed silver nanosheets, as well as their smaller average sheets size (ca. 42 nm as estimated from its XRD using Scherrer’s equation^14^) compared to that of lily-flower structures (with estimated average particle size of ca. 69 nm) obtained using higher malonic acid concentration. Attempts to use even lower concentrations down to 10 ppm, malonic acid resulted in disordered silver sheet-like structures (data not shown). It is clear that the acid induces formation of the nanosheets. The use of succinic acid (~50 ppm) as a directing agent resulted in the creation of chrysanthemum flower-like structures, which is mainly ball-shaped structures with short protrusions (see Fig. 1D, referred as Ag_chrysanthemums-flower_/GCE).

Interestingly, using higher succinic concentration (~100 ppm, notated as Ag_Rose-flower_/GCE, color code green) showed rose-flower silver like structures (see Fig. 1E) with much better electrocatalytic performance for hydrogen peroxide electrosensing. These results indicate the essential role of the directing agents (i.e., malonic and succinic acid) in controlling the silver particles morphology and roughness. However, the assembly of these nanostructures is significantly influenced by the added amount of the dicarboxylic acids as directing agents, the mechanism of such assembly is still unclear. For examples, very low concentration of succinic and malonic acids down to 10 ppm led to formation of non-assembled silver nanosheets structures, therefore, we suggest that the added acids might form complexes with the silver ions or adsorb on the surface of the silver nucleates and induce a new way of assembly leading to different morphology.

**Figure 2:**
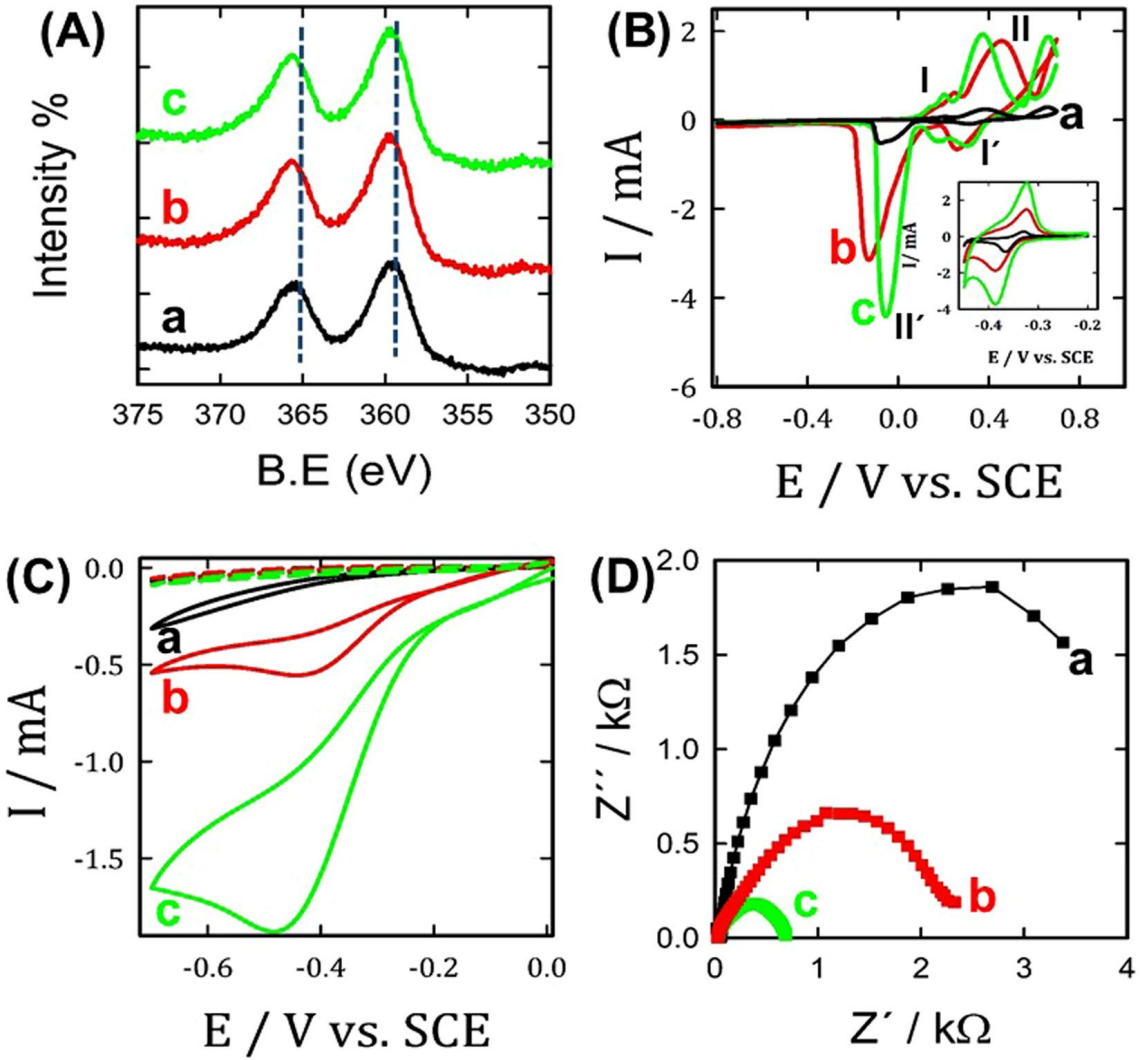
(**A**) High resolution Ag 3d XPS spectra of different studied silver microstructures; Ag_ball_ (black-line, curve a), Ag_lily-flower_ (red-line, curve b) and Ag_Rose-flower_ (green-line, curves c), (**B**) CVs obtained at same silver microstructures (same color code from Fig. 2A); Ag_ball_ (curve a), Ag_lily-flower_ (curve b) and Ag_Rose-flower_ (curve c) in 0.1 M KOH with a scan rate of 0.1 V/s (inset shows the Pb (UPD) stripping measured in 0.1 M HCl containing 10 mM Pb(NO_3_)_2_), (**C**) CVs measured at the same electrodes in the absence (dashed-lines) and in the presence of 1 mM H_2_O_2_ (solid-lines) in 0.1 M KOH with a potential scan rate of 0.02 V/s (same color code as in Fig. 2A. For E/V vs. SCE > 0, the graph mimic the response seen in panel A) and (**D**) Nyquist plots obtained at the same electrodes in 0.1 M KOH containing 1 mM H_2_O_2_ at −0.25 V vs. SCE (same color code as in Fig. 2A).

The crystallographic orientation and the crystallinity of the as-prepared silver catalysts were further investigated by XRD. As revealed in Fig. 1F, all the three tested silver catalysts (in absence and in the presence of malonic or succinic acids) exhibited the typical five diffraction peaks of the face-centered cubic (fcc) Ag crystal phase, these diffraction peaks can be indexed to the (111), (200, (220), (311), and (222) crystal planes of the as-prepared silver structures. Furthermore, using both directing agents led to smaller crystallite sizes as evidenced by their broader reflection peaks, besides, the directing agents existence resulted in smaller negative shift in all reflection peaks. This might be suggested electronic interaction between directing agents and silver nanoparticles which is confirmed next using XPS technique. *XPS spectra of the silver nanoparticles obtained in the absence of both directing agents (Ag*_*ball*_ *) and in the presence of 100 ppm of succinic acid and 100 ppm malonic acid were further investigated to determine if their presence might exert any electronic effect on the silver nanoparticles. As revealed in* Fig. 2A *, the presence of 100 ppm of succinic acid (curve c, green-color) or 100 ppm malonic acid (curve b, red-color) resulted in approx. 0.1 eV and 0.07 eV negative shift of the Ag3d doublet peaks compared to that of Ag*_*ball*_ *(curve a, black-line), respectively. This might be suggested strong electronic interaction between silver nanoparticles and the directing agents*.

Figure 2B shows the characteristic voltammetric (CVs) behavior of the three studied catalysts in 0.5 M potassium hydroxide (KOH). As seen in this figure, all of them exhibited the typical characteristic behavior of a clean polycrystalline silver electrode, where two main redox peak couples I/I´and II/II´ are observed in their respective cyclic voltammograms (CVs) attributed to the successive oxidation at the surface of the Ag particles from Ag to Ag^2+^ ^15^. Interestingly, the Ag_lily-flower_/GCE (using 50 ppm malonic acid, red-line, curve b) and Ag_Rose-flower_/GCE (using 100 ppm succinic acid, green-line, curve c) exhibited higher peak intensities (i.e., II/II´) compared to that of the Ag_ball_/GCE (black-line, curve a). This indicates that Ag_lily-flower_/GCE and Ag_Rose-flower_/GCE electrodes have a higher surface area compared to that of the Ag_ball_/GCE electrode.

The electrochemically active surface area of the as-prepared Ag microstructures was estimated by lead under potential deposition (UPD) stripping^15^ using the standard reported value of 400 μC/cm^2^ (see inset of Fig. 2B). The estimated active surface area was 0.9, 3.3 and 8 cm^2^ for the Ag_ball_/GCE, Ag_lily-flower_/GCE and Ag_Rose-flower_/GCE electrodes, respectively. That is, Ag_Rose-flower_/GCE exhibited 8 and 2.4 times higher active surface area compared to Ag_ball_/GC and Ag_lily-flower_/GC electrodes, respectively.

The electrocatalytic activity of the as-prepared catalysts towards hydrogen peroxide electrosensing was further investigated by CVs. Figure 2C shows the CVs of the Ag_ball_/GCE, Ag_Rose-flower_/GCE and Ag_lily-flower_/GCE electrodes in 0.1 KOH aqueous solution in the absence (dashed-lines) and presence of 10 mM H_2_O_2_ (solid-lines). As shown in this figure, in the absence of H_2_O_2_ (dashed-lines) only a small current was observed. The addition of 10 mM H_2_O_2_ resulted in a significant increase of the reduction current (solid-lines) attributed to the reduction of H_2_O_2_ according to the following equation:

$${H}_{2}{O}_{2}\Rightarrow {H}_{2}O+\frac{1}{2}{O}_{2}$$

Interestingly, Ag_lily-flower_/GCE (red-line) and Ag_Rose-flower_/GCE (green-line) electrodes exhibited outstanding activity compared to that of the Ag_ball_/GCE (black-line) electrode as evaluated by the significant positive shift of the onset potential together with the much higher current intensity of H_2_O_2_ reduction. That is, the Ag_Rose-flower_/GCE electrode showed 12 and 4 times higher electrocatalytic activity relative to that of Ag_ball_/GC and Ag_lily-flower_/GCE electrodes, respectively. This significant activity improvement of Ag_Rose-flower_/GCE electrode for H_2_O_2_ reduction is attributed to their higher surface area and unique flower-like structures.

It is worth to mention here that Ag_Rose-flower_/GCE (using 100 ppm succinic acid) exhibited higher activity and performance compared to Ag_chrysanthemums-flower_/GCE (using 50 ppm succinic acid), data not shown. Additionally, Nyquist plots (Fig. 2D) were measured at the as-prepared silver nanostructures which are obtained in the absence of both directing agents (Ag_ball_, black-line, curve a) and in the presence of 50 ppm malonic acid (Ag_lily-flower_, red-line, curve b) or 100 ppm succinic acid (Ag_Rose-flower_, green-line, curve c) to examine their charge transfer properties for hydrogen peroxide electroreduction. As shown in this figure, silver nanostructures (lily- and rose-flower structures) prepared using malonic and succinic acid as directing agents exhibited a lower charge transfer resistance compared to the silver nanoparticles obtained without using directing agents (ball-like structures).

**Figure 3:**
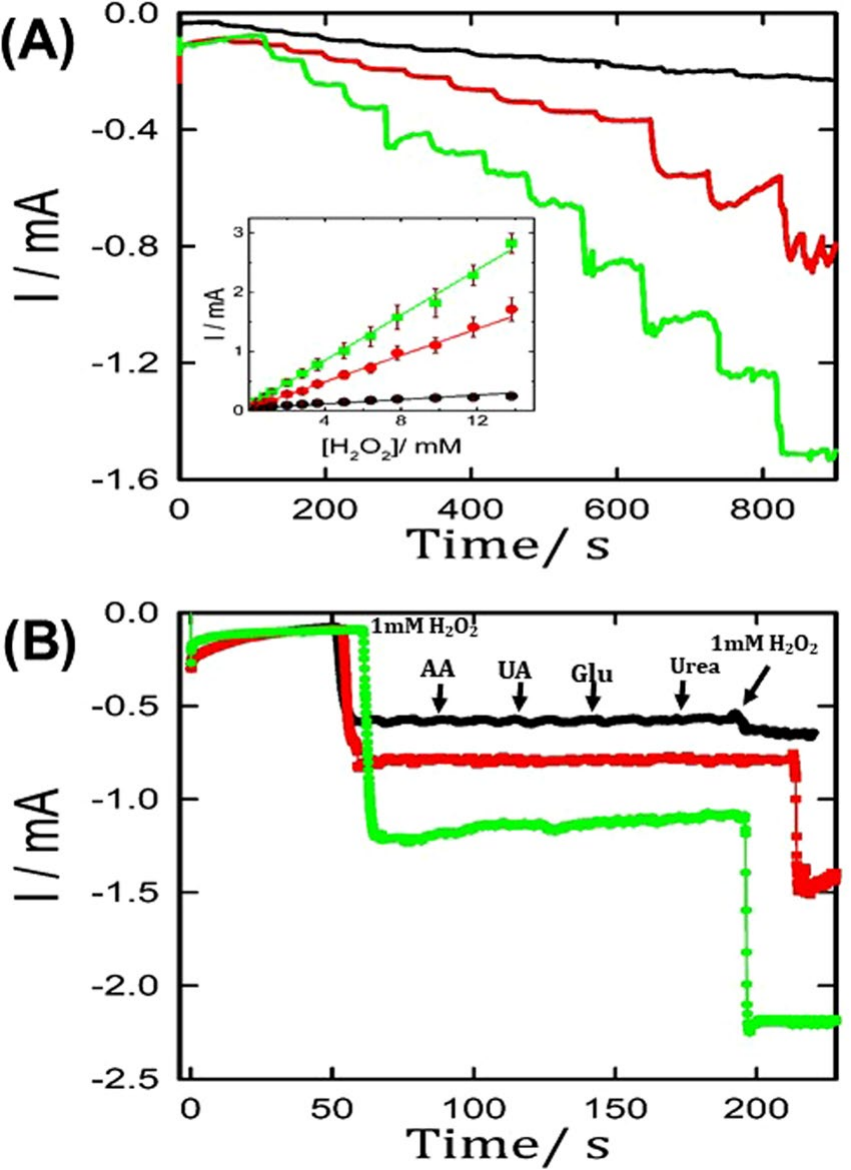
(**A**) Amperometric response (sensitivity test) obtained at silver microstructures in the absence (black-line) and presence of 50 ppm malonic (red-line) and 100 ppm succinic acid (green-line) with successive addition of H_2_O_2_ in N_2_-saturated 0.1 M KOH performed at −0.4 V. The inset shows the stepwise increase in the concentrations, which leads to the stepwise response of the current (mA), same colors code as in Fig. 3A). (**B**) Amperometric response (selectivity test) of the same electrodes after adding 1 mM H_2_O_2_, 0.1 mM ascorbic acid (AA), glucose (Glu), uric acid (UA) and Urea, respectively (same colors code as in Fig. 3A).

The H_2_O_2_ electrosensing performance (e.g., selectivity, durability and sensitivity) of the suggested silver microstructure catalysts was then evaluated by amperometric technique. Figure 3A shows the sensitivity test of the three studied silver microstructures. As clearly seen in this figure, the Ag_Rose-flower_/GCE electrode exhibited the best H_2_O_2_ electrosensing performance as demonstrated by its higher sensitivity with a rapid response time (2.4 mM mA^−1^ cm^−2^, 24 times higher), lower detection limit (0.4 μM, 16 times lower) together with higher linear range (up to 14 mM) compared to the Ag_ball_/GC electrode. Additionally, our proposed silver-based catalysts present a superior efficiency compared to various typical non-enzymatic H_2_O_2_ sensors (see Table 1). This enhanced H_2_O_2_ sensing efficiency at our proposed silver flower-like structures is attributed to their unique structures providing outstanding active surface areas with many hot-spot sites.

**Table 1:** A comparison of various non-enzymatic H_2_O_2_ sensors.

Sensors	Sensitivity (mA mM^−1^cm^−2^)	Detection limit (μM)	Linear range (mM)	References
Ag NW NT assembly	1.340	0.6	Up to 3.4	26
Ag NW colloid	0.00515	1.5	Up to 21.3	27
Ag NW colloid + graphene	0.01237	1.0	Up to 34.3	27
Electrodeposited Ag NW	0.374	334	Up to 10.1	28
Ag dendrites	0.0518	0.7	Up to 1.5	29
Ag-MnO_2_-MWCNTs/GCE	0.0825	1.7	Up to 10.4	30
Pt@Au NPS	0.3134	0.6	Up to 0.45	31
Pd core-Pt NDs-rGO	0.6728	0.03	Up to 0.1	32
Fe_3_O_4_/RGO	0.688	3.2	Up to 6.0	33
Ag_Rose-flower_/GCE	2.4	0.4	Up to 14	This work
Agball/GCE	0.022	1.8	Up to 14	This work
Ag_lily-flower_/GCE	0.480	1.0	Up to 14	This work

The interference-tolerance for the common interfering species (e.g., ascorbic acid, glucose, urea, uric acid) of the as-prepared silver-based catalysts was further investigated. As shown in Fig. 3B, all the studied silver microstructures-modified GCE electrodes exhibited an obvious amperometric response with the addition of 1 mM H_2_O_2_, while the successive addition of the above-mentioned interfering substances showed only a hardly discernible amperometric current response on the suggested sensors. This indicates the higher selectivity (i.e., high interference tolerance) of our proposed electrodes for non-enzymatic H_2_O_2_ sensing. Interestingly, both the Ag_lily-flower_/GC and Ag_Rose-flower_/GCE electrodes exhibited almost the same amperometric response of the first H_2_O_2_ addition after the second addition of H_2_O_2_ in the presence of the above-mentioned interfering species. On the other hand, the Ag_ball_/GCE electrode only displayed 15% from its original amperometric response, indicating a higher stability and interference tolerance of flower-shaped silver microstructures compared to the Ag_ball_/GCE electrode.

**Figure 4:**
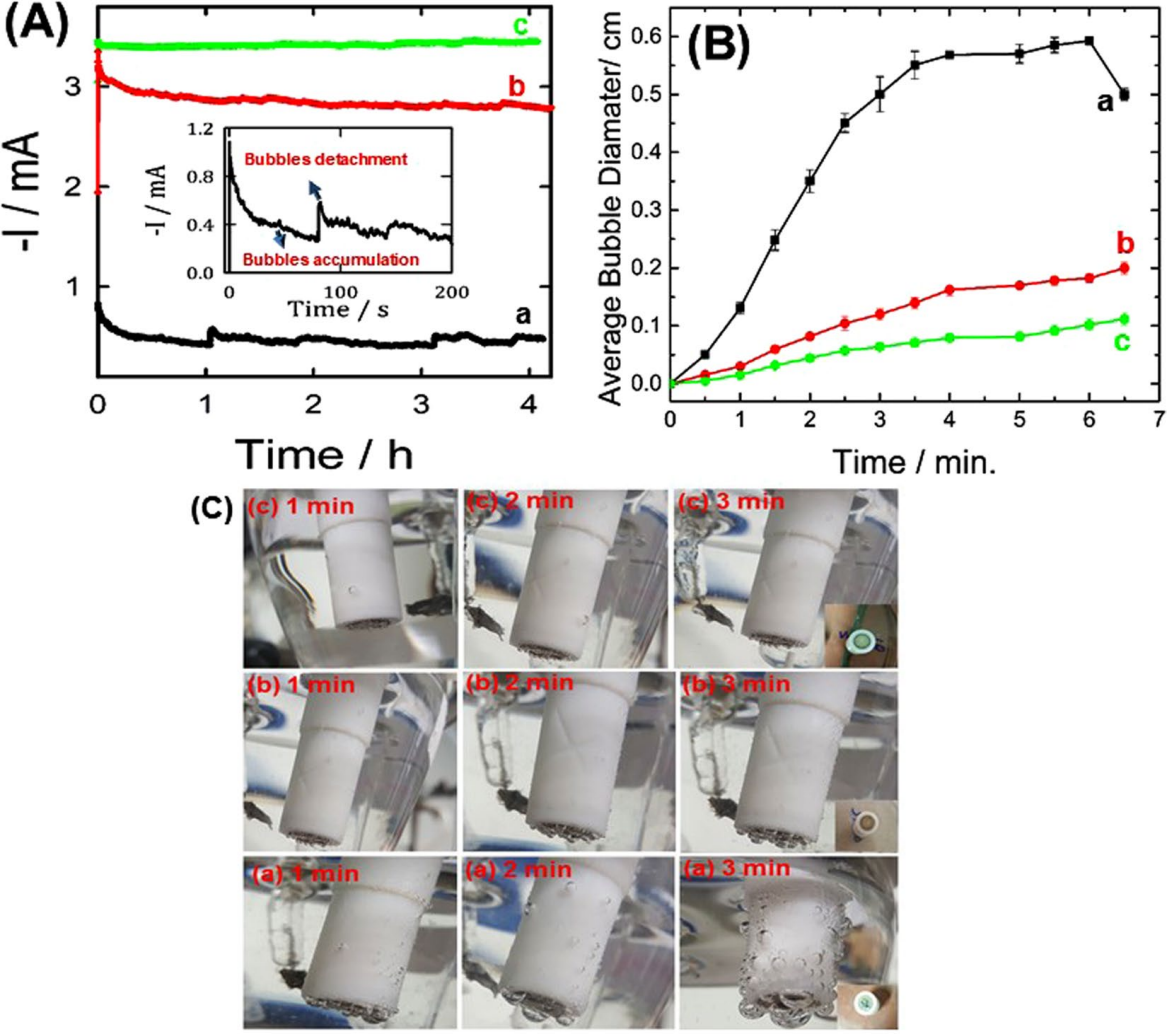
(**A**) current-transient (i–t) plots obtained at (a) Ag_ball_/GCE, (b) Ag_lily-flower_/GCE and (**c**) Ag_Rose-flower_/GCE E in 0.1 M KOH containing 5 mM H_2_O_2_ at −0.3 V vs. SCE. (**B**) variation of average bubble diameter with time of (a) Ag_ball_/GCE, (b) Ag_lily-flower_/GCE and (c) Ag_Rose-flower_/GCE and (**C**) the electronic images of the surface of the same electrodes at various times showing the bubble size and accumulation (same notations are used as in Fig. 4A, insets of these images show the surface of each electrode after measurement).

The current-transient curves (*i-t*) of all the studied silver-based sensors were performed in 0.1 M KOH containing 5 mM H_2_O_2_ at −0.3 V vs. SCE and the surface of these electrodes was imaged to estimate the effect of the *in-situ* formed oxygen bubbles size and their detachment rate on the as-prepared catalysts stability and performance, see Fig. 4. This figure shows the following interesting points:

- The catalytic stability of the Ag_ball_/GCE electrode decays rapidly with time (see Fig. 4A-black-line, curve a) by about 45% after only 20 minutes of continuous H_2_O_2_ electrolysis. The catalytic activities of both Ag_lily-flower_/GCE (red-line, curve b) and Ag_Rose-flower_/GCE (green-line, curve c) electrodes were reduced by only 8% and 12%, indicating the higher stability of the silver flower-like microstructures.
- The oxygen bubbles generated by H_2_O_2_ reduction atop of the Ag_lily-flower_/GCE are illustrated in panel b and Ag_Rose-flower_/GCE (image c) electrodes are about 6 times smaller compared to that of the Ag_ball_/GCE electrode (image a), see Fig. 4B. The rate of oxygen bubbles detachment from the Ag_lily-flower_/GCE and Ag_Rose-flower_/GCE surfaces is approximately 15 times faster compared that of the Ag_ball_/GCE electrode. When the oxygen bubbles accumulate atop of the Ag_ball_/GCE electrode and become larger, they block most of its surface-active sites. In other words, the rate of bubbles accumulation at the Ag_ball_/GCE electrode is much faster than their detachment rate. This can explain the high stability and activity of the Ag_lily-flower_/GC and Ag_Rose-flower_/GCE electrodes compared to that of the Ag_ball_/GCE electrode.
- As clearly seen in Fig. 4A, the stability of the Ag_ball_/GCE electrode rapidly decreases due to the bubbles accumulation on its surface blocking their active-surface sites, then it restores partially its activity due to the bubbles detachment (see the inset of Fig. 4A). This process is repeated as clearly revealed from the observed spikes and the images of the electrode surface. There were no spikes observed for the Ag_lily-flower_/GCE (Fig. 4A-curve c), indicating the higher bubbles detachment rate and their lower accumulation rate.

## Conclusion

Silver microstructures with different morphologies (e.g., flower- and ball-like structures) have been successfully prepared via a facile one-step precipitation method. The as-prepared silver flower-like microstructures exhibited outstanding performance as non-enzymatic H_2_O_2_ sensors. They show 24 times higher sensitivity (2.4 mM^−1^ cm^−2^) along with wider linear range (up to 14 mM) and smaller detection limit (about 0.4 µM, 16 times lower) compared to silver ball-like microstructures. This significant performance enhancement was attributed to their unique flower-like structures providing higher active surface area (8 times higher surface area) and facilitating the hydrogen peroxide reduction charge transfer together with the electronic interaction between the capping agents and silver nanoparticles. In addition, the suggested silver microflowers showed a much better stability about 11 and 3 times higher compared to that of silver nanoparticles with ball- and lily-flower like structures. Their higher stability was believed to originate from the smaller generated oxygen bubbles size and their faster detachment rates along with lower rate of bubbles accumulation on its surface relative to that of the silver ball-like structures.
